# Fecal microbiota transplantation from *Suncus murinus*, an obesity-resistant animal, to C57BL/6NCrSIc mice, and the antibiotic effects in the approach

**DOI:** 10.3389/fmicb.2023.1138983

**Published:** 2023-04-06

**Authors:** Mingshou Zhang, Hiraku Sasaki, Ting Yang, Juefei Chen, Rujia Li, Cheng Yi, Jun Li, Maozhang He, Shuang-Qin Yi

**Affiliations:** ^1^Department of Frontier Health Sciences, Graduate School of Human Health Sciences, Tokyo Metropolitan University, Tokyo, Japan; ^2^Department of Health Science, School of Health and Sports Science, Juntendo University, Bunkyō, Japan; ^3^Suzhou Center for Disease Control and Prevention, Suzhou, China; ^4^State Key Laboratory of Oncogenes and Related Genes, Shanghai Cancer Institute, Renji Hospital, School of Medicine, Shanghai Jiao Tong University, Shanghai, China; ^5^Department of Microbiology, School of Basic Medical Sciences, Anhui Medical University, Hefei, China

**Keywords:** fecal microbiota transplantation, antibiotics, obesity-resistant, gut microbiota, 16S rRNA, *Suncus murinus*

## Abstract

**Introduction:**

Important studies on the relationship of the intestinal microbial flora with obesity have uncovered profound changes in the composition of the gut microbiota in obese individuals. Animal studies successfully altered body phenotypes by fecal microbiota transplantation (FMT).

**Methods:**

In this study, we analyzed the gut microbiome of *Suncus murinus* (*S. murinus*), a naturally obesity-resistant animal, and the changes of the gut flora of C57BL/6NCrSIc mice that received gut bacteria transplantation from *S. murinus* by 16S rRNA gene analysis method. And analyzed and discussed the possible impact of the use of antibiotics before transplantation on the outcome of transplantation.

**Results:**

Our results showed no significant changes in body weight in the FMT group compared to the control (AB) group, but large fluctuations due to antibiotics. There was no change in blood lipid levels between groups before and after FMT. The gut microbiota of *S. murinus* were enriched in Firmicutes and Proteobacteria, while Bacteroidetes were not detected, and fewer OTUs were detected in the intestine gut in comparison to other mouse groups. Statistically significant differences in alpha diversity were observed between the FMT group and other groups. Furthermore, a beta diversity analysis indicated an apparent structural separation between the FMT group and other groups.

**Conclusion:**

It was suggested that the gut flora of *S. murinus* was not well established in the gut trace of mice through FMT, and the administration of antibiotics before transplantation was an important factor affecting the overall composition of the gut flora. Although FMT of *S. murinus* failed to completely colonize the intestinal tract of the mice, it still had a certain effect on the establishment of the intestinal flora of the mice. The unpredictable effects of pre-transplantation antibiotics on the results of transplantation cannot be ignored.

## Introduction

The human intestine harbors an enormously complex, diverse, and vast microbial community, is estimated to consist of at least 10^14^ bacteria and archaea, composed of approximately 1,100 prevalent species, with approximately 160 such species per individual. The microflora is estimated to contain 150-fold more genes than our own host genomes (Qin et al., 2010). The gut microbiome plays a role in the regulation of weight and metabolism by increasing energy extraction from food, altering energy expenditure, and modulating appetite and satiety, glucose homeostasis, and lipid metabolism in humans (Clarke et al., 2014; Leong et al., 2018). Important studies on the relationship of the intestinal microbial flora with obesity have uncovered profound changes in the composition and metabolic function of the gut microbiota in obese individuals (Ley et al., 2005; Turnbaugh et al., 2007; Tilg and Kaser, 2011).

Recently, research on the modulation of the gut microbiota has focused on fecal microbiota transplantation (FMT), which refers to a method of transfer of feces from a healthy individual to the gut of a diseased recipient *via* an enema, endoscopy, nasogastric tube, or by indigestion peroral capsules (Takáčová et al., 2022). It is considered an innovative, effective, and safe technique to treat challenging diseases like *Clostridium difficile* (Bárcena et al., 2019), *Campylobacter jejuni* (Brooks et al., 2017; Heimesaat et al., 2019). Animal studies successfully altered body phenotypes by FMT. Germ-free mice that received microbiota from human donors with obesity developed obesity, whereas mice that received microbiota from human donor post bariatric surgery remained lean (Ridaura et al., 2013; Tremaroli et al., 2015; Leong et al., 2018, 2020). This evidence highlights that FMT could be a treatment modality for human obesity (Ridaura et al., 2013; Tremaroli et al., 2015). Furthermore, there are also studies of the effects of antibiotic treatment that transfer the gut microbiota from lean mice into obese mice (Ellekilde et al., 2014). In order to reduce the interference of the intestinal flora of the transplant receipt on the colonization of the transplanted flora, it is necessary to use antibiotics before FMT, but the use of this program in the current literature is not uniform (Supplementary 1), and researchers have rarely discussed the impact of antibiotic use on the transplant itself.

The house musk shrew (*Suncus murinus*), is an insectivorous animal that lives in houses and grassy areas near human habitations or in cattle pens, and is widely distributed throughout Asia and East Africa (Yi et al., 2003, 2005). As a laboratory animal, we have continued research on the morphological analysis of nerves and the gastrointestinal tract and the development of pathological models (Yi et al., 2006, 2007). *S. murinus* has unique gastrointestinal tract characteristics: a very simple structure (with no fermentative chambers), a very short total intestine length, and a very short large intestine length (Yi et al., 2011). In our recent studies, we investigated the obesity-resistance phenomenon in *S. murinus*. It was found that the weight of *S. murinus* did not change much after 2 months of age, and that mesenteric fat did not accumulate after birth (Yi et al., 2010; Zhang et al., 2020). Thus, *S. murinus* may be a suitable model for investigating obesity and metabolic syndrome, particularly the mechanism of obesity resistance (Yi et al., 2010). Aiming to continue research on this natural obesity-resistant animal, we focused on the microbes that inhabit their intestines.

In this study, we performed an experimental study of FMT between *S. murinus* (as donors) and C57BL/6NCrSIc mice (as recipients). The microbiota of donor *S. murinus* was analyzed and the changes of the body weight and blood lipid level in the C57BL/6NCrSIc mice groups were evaluated. Furthermore, the microbiota changes among C57BL/6NCrSIc mice groups were compared to evaluate FMT from *S. murinus* and the effects of a cocktail of broad-spectrum antibiotics.

## Methods

### Animals

#### Suncus murinus

Male house musk shrews (*S. murinus*) (*n* = 5; age, 4 weeks), which were obtained from a closed breeding colony (JIc: KAT-c, at our laboratory) were employed as a donor (donor control group, DC group) for the following the fecal microbiota transplantation (FMT) experiment (Dai et al., 2019; Zhang et al., 2020). All animals were housed in polycarbonate cages in a room maintained at 28 ± 2°C with 50 ± 5% relative humidity in the Functional Morphology Laboratory, Department of Frontier Health Sciences, Tokyo Metropolitan University, Japan. *S. murinus* were randomly swapped between cages 3 times per week for 3 weeks prior to the FMT procedure to minimize other variables causing differences in the microbiota (McLeod et al., 2018). The room was automatically lit between 09:00 h and 21:00 h. The pellets consisted of 45.0% protein, 4.0% fat, 3.0% fiber, 15.0% ash and 26.2% complex carbohydrate (Oriental Yeast Co., Ltd. Bioindustry Division, Chiba, Japan), and metabolizable energy content was 357 kcal/100 g; and pellets and water were supplied *ad libitum.*

#### C57BL/6NCrSIc mouse

Specific pathogen free (SPF) male C57BL/6NCrSIc mice (male, *n* = 15; age, 4 weeks) obtained from Sankyo Labo Service Corporation (Inc. Tokyo, Japan) were housed in high-efficiency, particulate air-filtered cages with sterilized bedding with *ad libitum* access to food (standard chow, Nosan Corporation Laboratory Animal Feed, Kanagawa, Japan). The pellets consisted of 18.8% protein, 3.9% fat, 6.6% fiber, 6.9% ash, and 54.7% complex carbohydrate, and metabolizable energy content was 373 kcal/100 g; and pellets and water were supplied *ad libitum.* All mice were kept on a 12 h light/dark cycle at 25 ± 2°C in our laboratory.

### Experimental design and procedure

All animal experiments were approved by the Institutional Animal Care and Use Committee of Tokyo Metropolitan University and all experiments were conducted in accordance with the National Research Council Guide for Care and Use of Laboratory Animals (A2-2, A3-22, A4-19).

**Figure 1:**
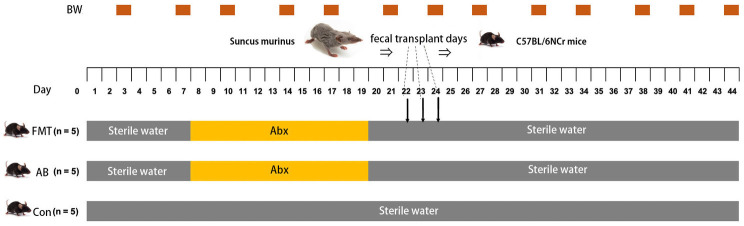
Schedule of antibiotic treatment period (Abx), fecal microbiota transplantation (FMT) and body weight measurements (BW). FMT, FMT group; AB, antibiotic group; Con, Control group.

As shown in Figure 1, mice were randomly divided into three groups, the control group (Con group, *n* = 5), the antibiotics control group (AB group, *n* = 5), and the FMT group (*n* = 5). The FMT group received FMT from *S. murinus* three times after treatment with antibiotics in the third week. The AB group was treated with a combination of antibiotics without receiving transplantation. The Con group did not receive antibiotic treatment or transplantation. To remove indigenous gut microorganisms, we referred to previous reports on antibiotic administration (Chen et al., 2020; Gudi et al., 2020; Su et al., 2020). The antibiotics included a combination of ampicillin (1.0 g/L; Nacalai Tesque, Kyoto, Japan), vancomycin (0.5 g/L; Shionogi, Osaka, Japan), neomycin (1.0 g/L; Nacalai Tesque, Kyoto, Japan), and metronidazole (1.0 g/L; Nacalai Tesque, Kyoto, Japan), which was added to drinking water for 10 consecutive days to remove indigenous gut microorganisms. After a 3-day recovery period, FMT was performed on the next 3 consecutive days.

### Fecal microbiota transplantation

To collect fecal content from the donor animal, first, all surgical instruments were aseptically sterilized before transplantation surgery. Age and sex-matched donor animals (*S. murinus*) were deeply anesthetized; then, the abdominal skin was disinfected with iodine, midline abdominal incision. The large intestine was cut open, 300 mg of the content was collected, 200 mg of it was transferred to 1.5 mL sterile test tube and stored at −80°C for DNA extraction, and another 100 mg was transferred into a 5 mL sterile test tube with care taken to avoid external contamination. We modified Marino’s protocol (McLeod et al., 2018) slightly as follows: 100 mg of fecal content was collected and resuspended with 1 mL of sterile phosphate-buffered saline (pH = 7.2) (Containing 10% triglyceride) in 1.5 mL of sterile microtube, then the microtube was vortexed for 10 s and centrifuged at 805 × *g* for 3 min at 4°C to separate the supernatant that contained most of the microbiota from the solid fecal matter. Approximately 600 μL of supernatant was divided into 3 equal parts (200 μL/per tube), with 1 vial immediately available for transplant experiments and the other two stored at −80°C in a freezer for use over the next 2 days. Intestinal content supernatant (200 μL) was administered by oral gavage for 3 days. At 3 weeks after transplantation, the dose of anesthesia was increased, and the donor animal was sacrificed. The mice in the Con and AB groups, which did not receive FMT equally received oral gavage of PBS in order to match the stress of gavage manipulation during the same 3-day period.

### Body weight and blood lipid level

The body weight was measured twice a week (Figure 1). To measure the blood lipid level, 500–1,000 μL of blood was collected from the jugular vein of each mouse on the day before FMT and the last day of the experiment, and centrifuged at 3,000 rpm for 10 min at 4°C, and then the blood serum samples were stored at −20°C until next use. The blood lipid levels for total cholesterol (T-Cho) (Co. 439-17501; Wako Inc., OSA, Japan), triglycerides (TG) (Co. 432-40201; Wako Inc., OSA, Japan), and phospholipids (PL) (Co. 433-36201; Wako Inc., OSA, Japan) were analyzed using an automatic chemistry analyzer according to the manufacturer’s protocol (Zhang et al., 2020).

### Extraction of DNA from feces

After the mice were anesthetized to a state of lethargy, under operating conditions that were aseptic as possible, the abdominal cavity of the mouse was opened, the cecum was cut open, 200 mg of feces was collected, then the collected feces were immediately dissolved with the reagents in the DNA Kit and fecal DNA was extracted from fecal samples using an ISOSPIN Fecal DNA Kit (NIPPON GENE CO., LTD, Japan) according to the manufacturer’s protocols. A NanoDrop 2000 spectrophotometer (Thermo Scientific, United States) was used to detect the concentration of the extracted DNA. Any samples that did not meet the detection standards were removed. All DNA samples were stored at −80°C immediately before the next process.

### Library construction and sequencing

The paired-end (2 × 300 bp) sequencing was performed by the Macrogen (South Korea) using the MiSeq^™^ platform (Illumina, San Diego, United States). It was completed with reference to the report of Baek et al. (2022). The details were as follows. The sequencing libraries were prepared according to the Illumina 16S Metagenomic Sequencing Library protocols to amplify the V3–V4 region. The input gDNA 2 ng was PCR amplified with 5× reaction buffer, 1 mM of dNTP mix, 500 nM each of the universal F/R PCR primer, and Herculase II fusion DNA polymerase (Agilent Technologies, Santa Clara, CA, United States). The 1st PCR cycle conditions were as follows: 3 min at 95°C for heat activation, and 25 cycles of 30 s at 95°C, 30 s at 55°C and 30 s at 72°C, followed by a 5-min final extension at 72°C. The universal primer pair with Illumina adapter overhang sequences used for the first amplifications were as follows: V3-F: 5′-TCGTCGGCAGCGTCAGA TGTGTATAAGAGACAGCCTACGGGNGGCWGCAG-3′, V4-R: 5′- GTCTCGTGG GCTCGGAGA TGTGTATAAGAGACAGG ACTACHVG GGTATCTAATCC-3′. The 1st PCR product was purified with AMPure beads (Agencourt Bioscience, Beverly, MA, United States). Following purification, the 2 μL of the 1st PCR product was PCR amplified for final library construction index using NexteraXT Indexed Primer. The 2nd PCR cycle conditions were the same as the 1st PCR conditions, except that there were 10 cycles. The PCR product was purified with AMPure beads. The final purified product was then quantified by qPCR according to the qPCR Quantification Protocol Guide (KAPA Library Quantification kits for Illumina Sequencing platforms) and qualified using TapeStation D1000 ScreenTape (Agilent Technologies, Waldbronn, Germany).

### Sequence analysis

For adapter trimming, the adapter sequences were removed, and error-correction was performed for areas where the two reads overlapped using fastp (v. 0.19.7) (Chen et al., 2018). Subsequently, the original library and single long reads were obtained by assembling paired-end sequences created by sequencing both directions of library using the FLASH software program (v.1.2.11) (Magoč and Salzberg, 2011).

Operational taxonomic units (OTUs) were clustered with the cut-off value set at 97% similarity and chimeric sequences were identified and removed using CD-HIT-OTU (cd-hit-otu-illumina-0.0.1)^1^ (Li et al., 2012). A BLAST+(v2.9.0) search (Query coverage > 85% and identity > 85%) was performed for each 16S rRNA gene sequence against the RDP release 11 database (RDP Release 11 Update 4: May 26) to obtain taxonomy information.

### Bioinformatics and statistical analysis

The QIIME (V.1.9.1) (Caporaso et al., 2010) and R Language (version 3.4.4)^2^ were used for the sequencing analysis of the gut microbiota. Statistical differences in alpha diversity indices (i.e., the Shannon index, the inverse Simpson index, observed richness, Chao’s estimated richness (Chao 1), and the relative abundance of the different taxonomic groups) were measured from Krusal-Wallis test and Wilcoxon *t*-test. Furthermore, the bacterial community diversity was analyzed using rarefaction plots and Boxplots, and displayed using the R software program (version 3.4.4). Beta diversity was measured using Bray–Curtis dissimilarity, Unweighted unifrac, Weighted unifrac, and jaccard distance metrices. The tests for microbial community composition dissimilarity between pairs of groups were performed using nonparametric multi-response permutation procedures (MRPPs), analysis of similarities (ANOSIM), and nonparametric permutational multivariate ANOVA with the adonis function (Adonis) (Oksanen et al., 2022; RDocumentation, 2022) in R (version 3.4.4). A principal coordinates analysis (PCoA), based on the Bray-Curtis distance matrix, was used to perform in R (version 3.4.4) and PCoA figure was created using R package “ggplot2.” Distance-based methods, such as the (un) weighted pair group method with arithmetic mean (UPGMA) (Lozupone and Knight, 2005) have been used to conduct cluster analyses based on the similarity and dissimilarity of bacterial communities in samples. Heatmaps (Heatmap of differential species abundance clustering) were created using “pheatmap” R-package, Unique OTUs and those shared between samples were illustrated using a Venn diagram that was created in the R package “Venn Diagram” (Oksanen et al., 2022).

The two-sided t-test were used for the statistical analysis using R (version 3.4.4) and results were presented as the mean with the standard error of mean (SEM) and *p* values of < 0.05 were considered statistically significant.

## Results

No deaths or infectious complications from FMT were observed in this study.

### Body weight changes in each experimental group

In comparison to the Con group, the body weight of the AB and FMT groups decreased significantly after antibiotics administration; however, the body weight began to recover at 3 days after antibiotics were withdrawn. There was still a difference in body weight between the AB and Con groups (*p* < 0.05) or the FMT and Con groups (*p* < 0.05) until the end. There was no significant difference in body weight between the AB and FMT groups (*p* > 0.05) (Supplementary 2).

### Blood lipid level changes among those groups before and after FMT

The serum phospholipid, triglyceride and total cholesterol concentrations were not significantly changed in the FMT group compared with the Con and AB groups before (*p* > 0.05) and after (*p* > 0.05) the FMT procedure (Supplementary 3).

### Overview of the sequencing data

**Figure 2:**
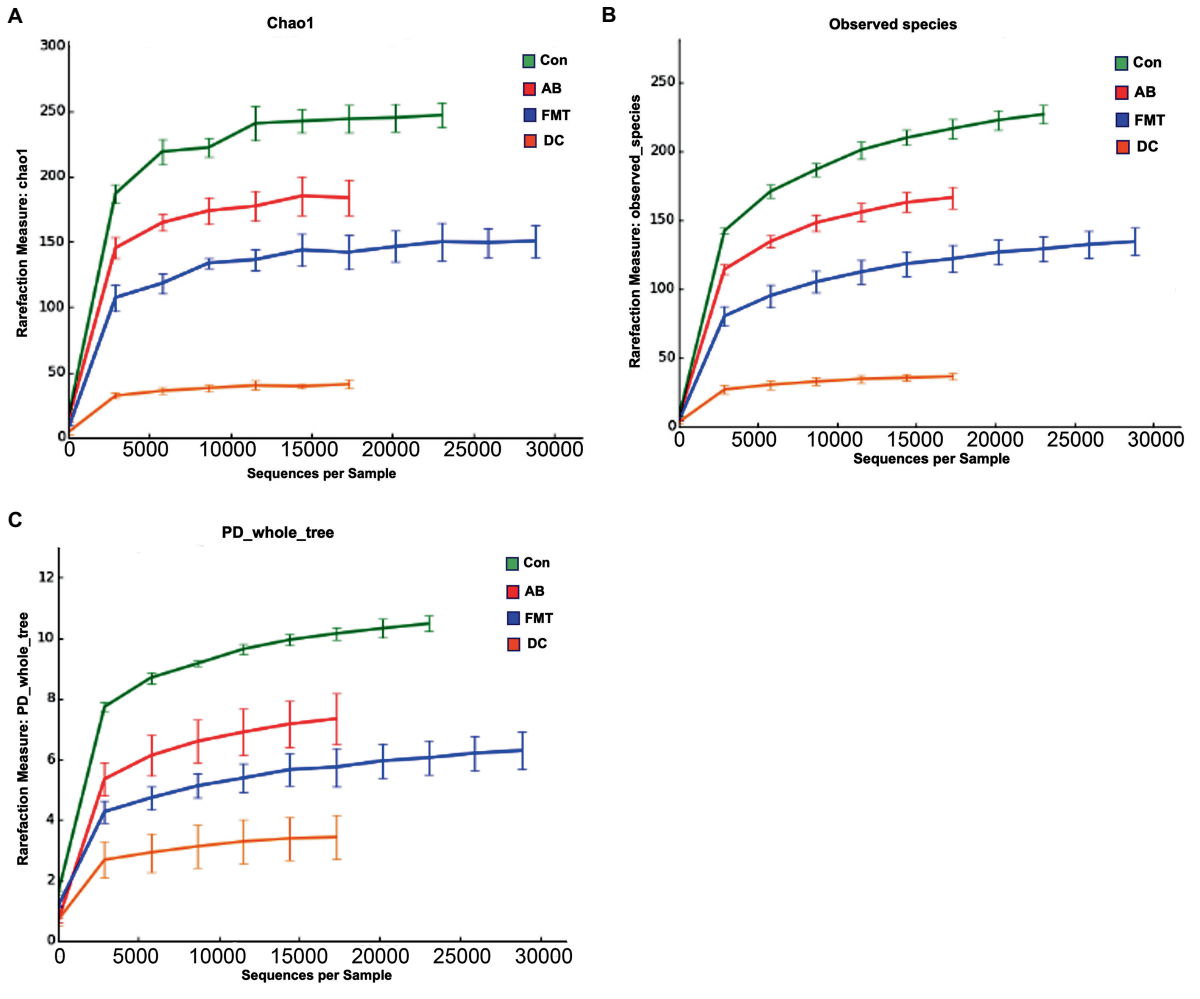
The species rarefaction curve of each experimental group. This graph represents the numbers of observed species under different sequences numbers extracted randomly, Chao 1 **(A)**, Observed species **(B)**, and PD whole tree **(C)**. Chao 1, Chao’s estimated richness; PD, Phylogenetic distance; Con, Control group; FMT, fecal microbiota transplantation group; AB, antibiotic group; DC, donor group.

In total, after filtering for chimeric and low-quality OTUs, 589,396 high-quality reads corresponding to 568 OTUs were identified, and 98 genera were annotated. The alpha diversity of the microbial communities was assessed and calculated (Table 1). Rarefaction curves (the phylogenetic diversity whole tree index, the Chao1 index, and the observed species) (Figure 2) of OTUs richness were calculated using the vegan library in the R statistical computing language (Oksanen et al., 2022). The rarefaction curves showed the number of species/OTUs under different sequence numbers, showing that the gut microbiota species/OTUs of the FMT group were significantly lower in comparison to the Con group (*p* < 0.01) or the AB group (*p* < 0.01), but significantly higher in comparison to the DC group (*p* < 0.01) (Figure 2).

**TABLE 1:** Diversity and richness (mean ± SD) of the fecal bacteria communities of mice and *Suncus murinus*.

Group	OTUs	Chao 1	Shannon	Inverse Simpson	Good’s Coverage
FMT	140.8 ± 13.53	159.32 ± 20.74	2.823352 ± 0.35	0.6602326 ± 0.06	0.9994487 ± 0.00
AB	177 ± 8.86	188.50 ± 15.45	4.582651 ± 0.42	0.901082 ± 0.05	0.9989312 ± 0.00
Con	233.8 ± 8.75	251.74 ± 13.81	5.08195 ± 0.12	0.9328051 ± 0.01	0.9989994 ± 0.00
DC	39.6 ± 4.03	42.05 ± 4.61	1.994455 ± 0.65	0.5864854 ± 0.21	0.9998596 ± 0.00

OTUs, operational taxonomic unit; Chao 1, returns the Chao 1 richness estimate for an OTU definition; Shannon, the Shannon index takes into account the number and evenness of species; Inverse Simpson, the Inverse Simpson index represents the probability that two randomly selected individuals in the habitat will belong to the same species; Good’s Coverage, Coverage is calculated as *C = 1-(s/n)*. Con, Control group; FMT, fecal microbiota transplantation group; AB, antibiotic group; DC, donor group.

### Bacteria composition and relative abundance

A total of 11 phyla, 19 classes, 30 orders, 54 family, 98 genus and 568 species were detected in the prokaryotic microbiota communities from all fecal samples.

**Figure 3:**
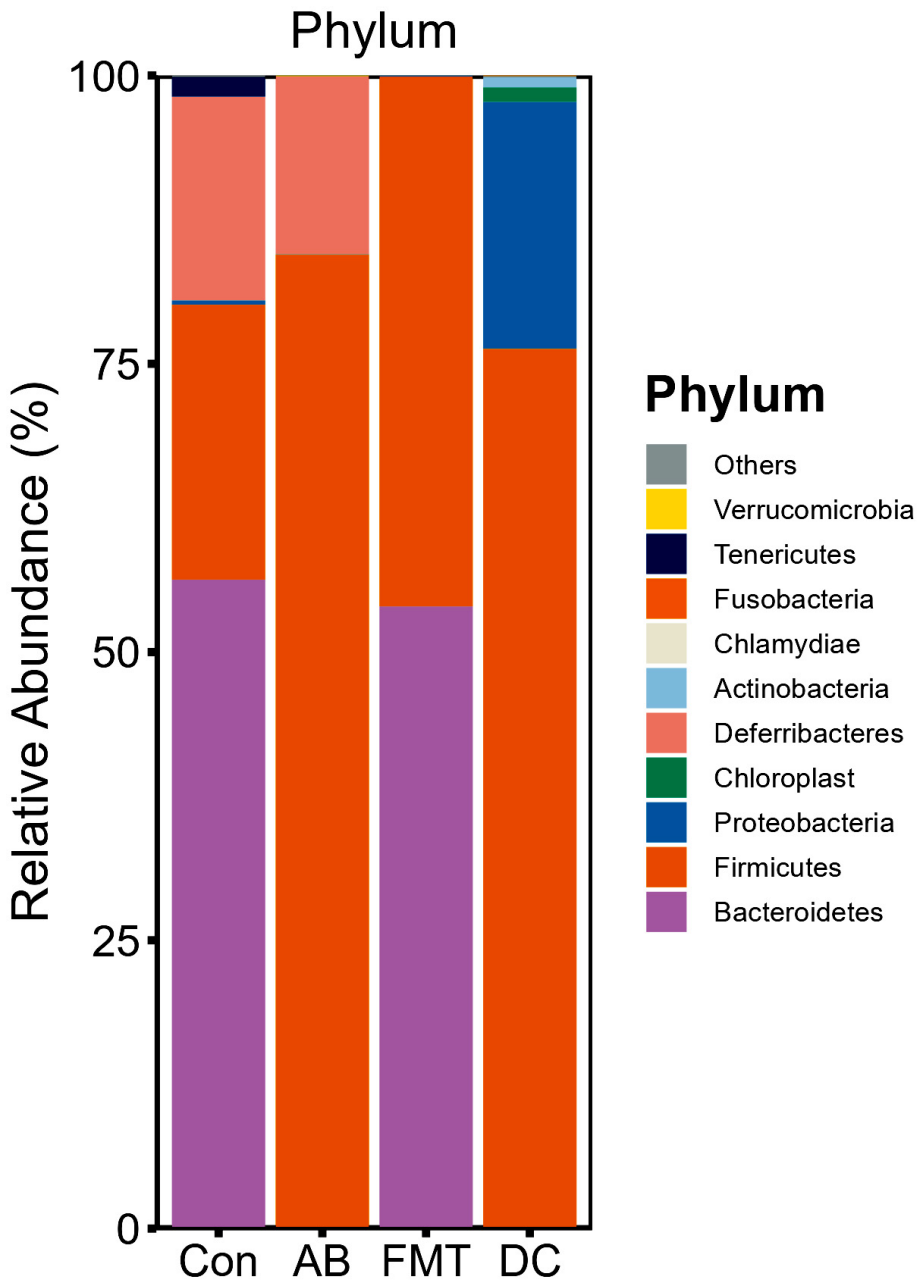
Relative abundance ratio of the intestinal microbiome at the phylum level in each experimental group. Data are presented as the percentage of species, and the results were obtained using the Kruskal–Wallis test. Con, Control group; FMT, fecal microbiota transplantation group; AB, antibiotic group; DC, donor group.

**Figure 4:**
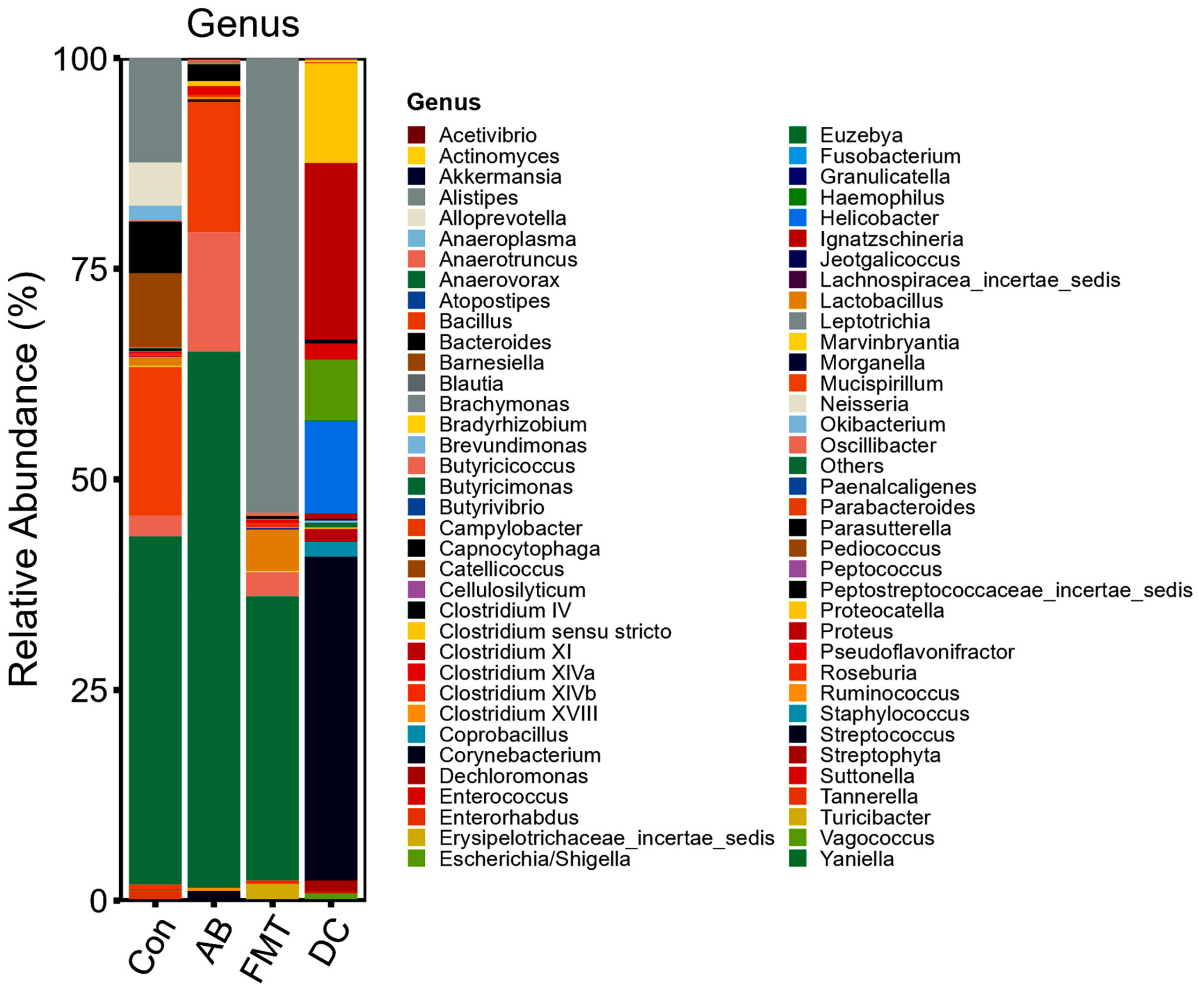
Relative abundance ratio of the intestinal microbiome at the genus level in each experimental group. Data are presented as the percentage of species, and the results were obtained using the Kruskal–Wallis test. Con, Control group; FMT, fecal microbiota transplantation group; AB, antibiotic group; DC, donor group.

We chose the main phyla and genera based on species abundance to generate a histogram, which showed the percentages of relative abundance in each sample or group (Figures 3, 4).

At the phylum level (Figure 3), the top five ranked abundance-based phyla were Bacteroidetes (55.89%), Firmicutes (24.06%), Deferribacteres (17.78%), Tenericutes (1.77%), Proteobacteria (0.37%) in the Con group. Intriguingly, the most abundant phyla were Bacteroidetes (54.46%) and Firmicutes (45.46%), and the abundant radio of the other phyla was <1% in the FMT group, while the most abundant phyla in the AB group were Firmicutes (85.62%) and Deferribacteres (14.16%), and the other phyla accounted for <1%. In summary, the most predominant phyla in all groups (Con, FMT, and AB) were Firmicutes, while the Deferribacteres disappeared in the FMT group and there were only a few Bacteroidetes in the AB group. For the donor *S. murnus*, the most abundant phyla were Firmicutes (75.55%) and Proteobacteria (21.07%); the other phyla accounted for <1%.

At the genus level (Figure 4), most genera in the FMT group were *Alistipes* (54.44%), *Lactobacillus* (4.83%), *Turicibacter* (1.94%), and *Oscillibacter* (2.94%). In the AB group, most genera were *Oscillibacter* (14.41%), *Mucispirillum* (14.16%), *Clostridium IV* (2.07%), and *Streptococcus* (1.07%). In the Con group, most genera were *Mucispirillum* (17.78%), *Alistipes* (12.38%), *Barnesiella* (8.71%), *Bacteroides* (6.04%), *Alloprevotella* (5.03%), *Oscillibacter* (2.39%), and *Lactobacillus* (1.03%). For the donor *S. murinus*, the most abundant genera were *Streptococcus* (35.26%), *Clostridium XI* (24.76%), *Helicobacter* (11.27%), *Clostridium sensu stricto* (10.06%), *Escherichia/Shigella* (6.61%), *Streptophyta* (2.0%), *Staphylococcus* (1.61%), *Enterococcus* (1.51%), *Lactobacillus* (0.68%), and *Vagococcus* (0.59%).

**Figure 5:**
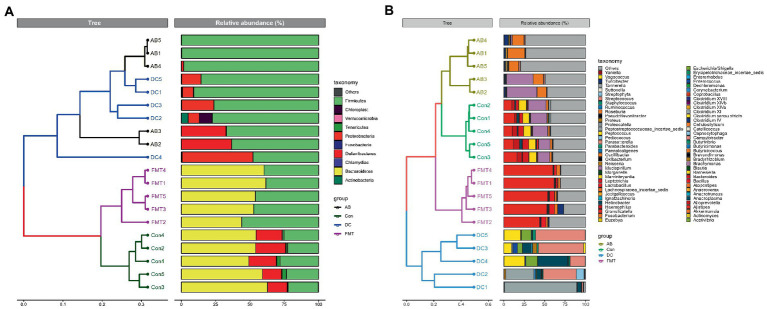
A Cluster analysis based on the Unifrac distance matrix **(A)** and on the weighted Unifrac distance **(B)** in groups at the phylum and genus levels. Control group: Con1, Con2, Con3, Con4, Con5. Antibiotic group: AB1, AB2, AB3, AB4, AB5. Decal microbiota transplantation group: FMT1, FMT2, FMT3, FMT4, FMT5. DC group: DC1, DC2, DC3, DC4, DC5.

A cluster analysis using the weighted Unifrac distance matrix was conducted after the UPGMA clustering method (Figure 5). The results were basically consistent with those from the clustering analysis using the unweighted Unifrac distance matrix (Supplementary 4).

### The analysis of discrepancies between groups

#### The alpha diversity analysis

**Figure 6:**
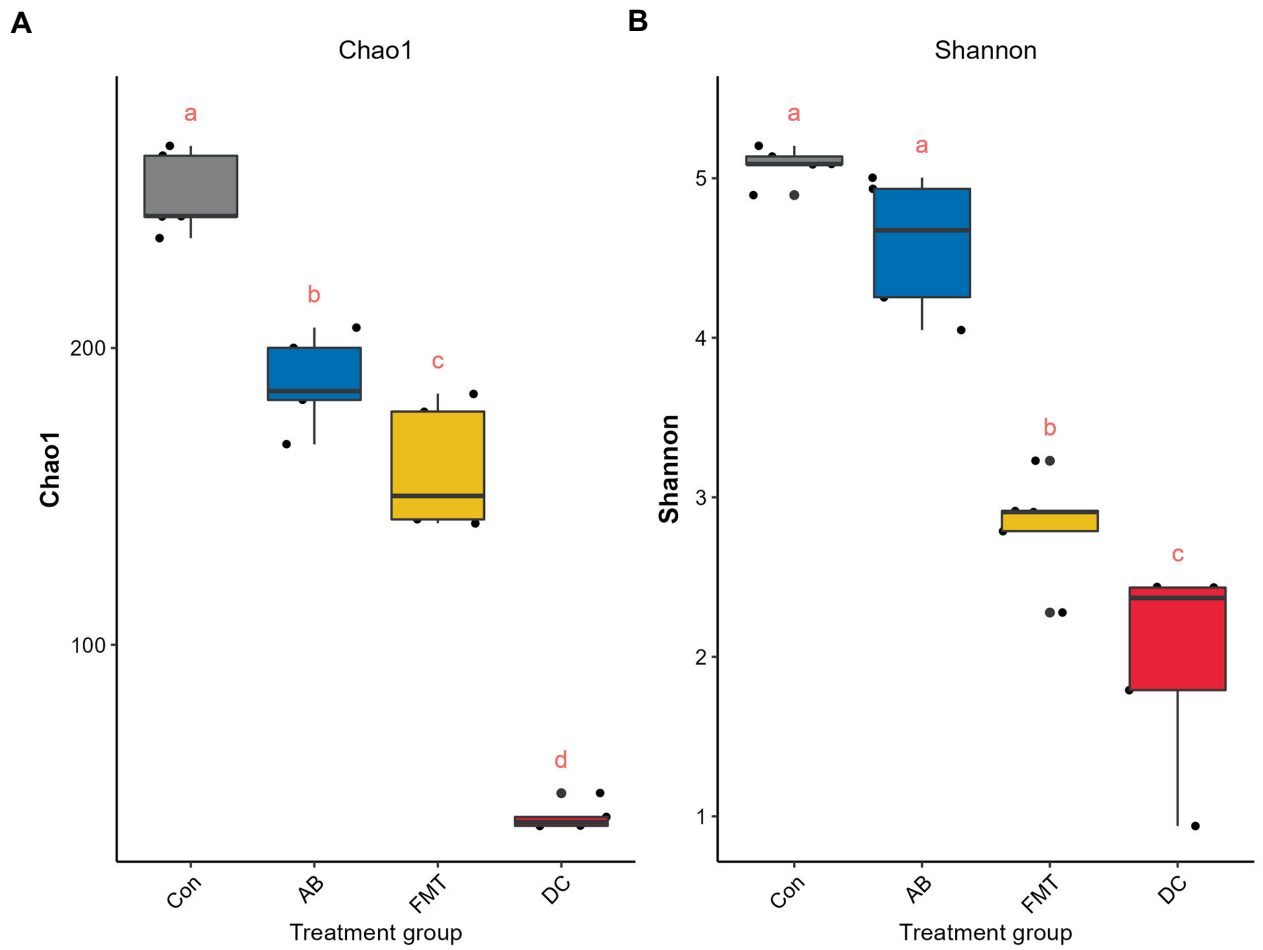
Comparisons of the alpha diversity, Chao 1 **(A)** and Shannon **(B)** index. The same letter indicates that there was no significant difference between the groups; a different letter indicates a statistically significant difference between the groups. Con, Control group; FMT, fecal microbiota transplantation group; AB, antibiotic group; DC, donor group.

To analyze the overall microbial community structure of the donor and mouse groups, we used four different measures of diversity index: Chao 1, Shannon diversity index, Inverse Simpson, and OTUs, to determine the alpha diversity (Table 1). Wilcoxon’s tests at the genus level were used to calculate the alpha diversity (Shannon and Chao 1) (Figure 6). The comparison of α diversity showed that there was a significant difference in species abundance (Chao 1) or Shannon index among those groups. The gut microbiota of the FMT group featured a markedly decreased Shannon index (Figure 6B and Table 1) and Chao1 index (Figure 6A and Table 1) in comparison to the Con or AB groups, while the diversity and richness of the FMT group were significantly higher than those in the DC group. The Shannon diversity index (*p* < 0.001) (Figure 6B and Table 1), and Chao 1 index (*p* < 0.001) (Table 1) showed that the Con group had significantly higher richness in comparison to the other groups. While the diversity in the Con group showed no significant difference in comparison to the AB group.

#### The beta diversity analysis

**Figure 7:**
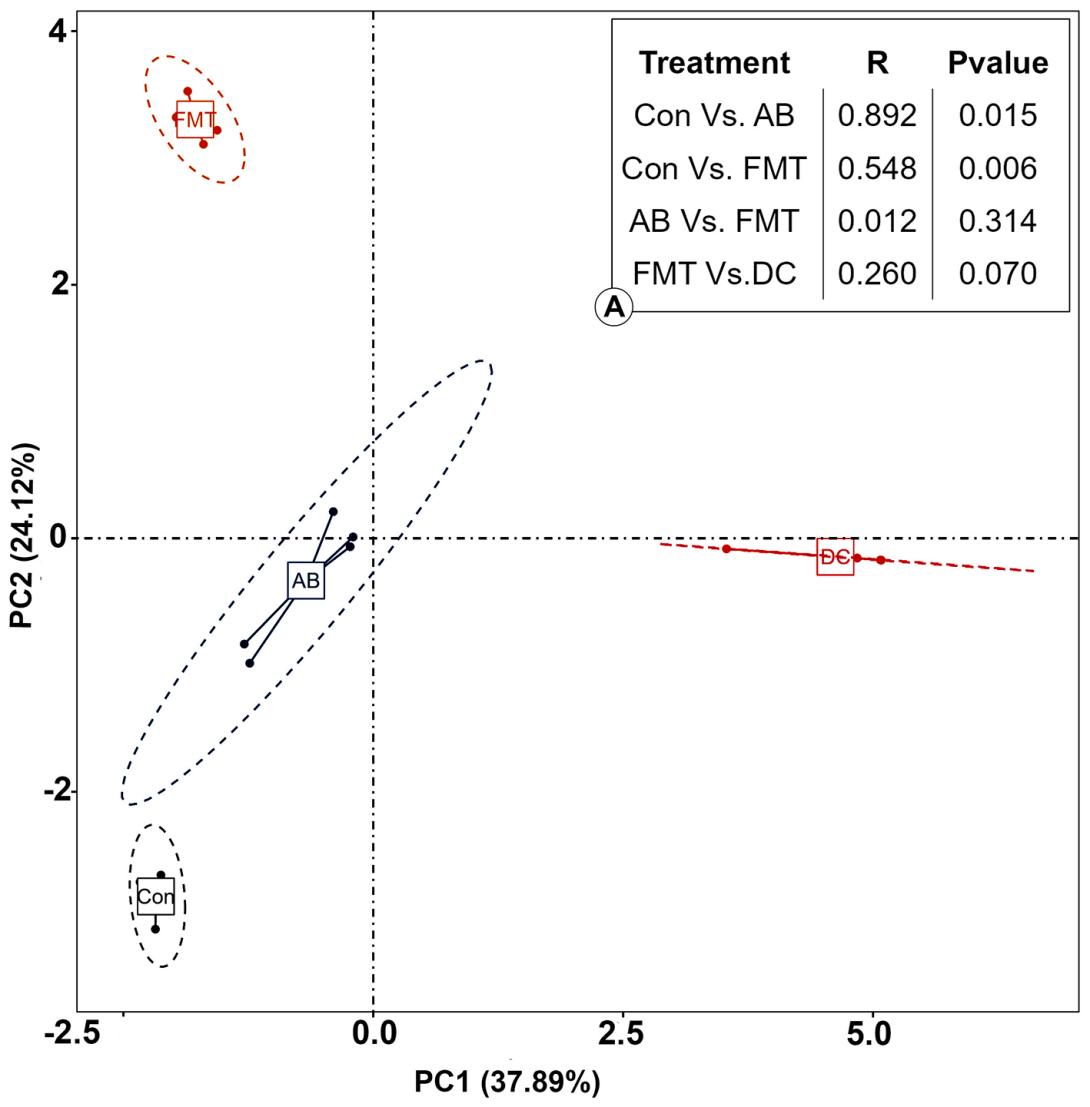
The principal coordinate analysis (PCoA) of the bacterial community structures of mice and donor gut microbiomes based on the Bray-Curtis (weighted UniFrac) distance. (A): The analysis of similarity (ANOSIM) of the four groups. Con, Control group; FMT, fecal microbiota transplantation group; AB, antibiotic group; DC, donor group.

The analysis of similarity (ANOSIM) (Figure 7) and multi-response permutation procedures (MRPP) (Table 2) indicated significant differences in the bacterial communities between fecal samples collected from different groups. To compare the similarity between samples and groups, a PCoA was used to clarify the similarity of the bacterial communities in the samples collected from different groups (Figure 7). Visualization of all sequenced samples using two-dimensional PCoA revealed clear site-specific clustering with different treatments. Combination with ANOSIM revealed that the gut microbiota in the Con group was significantly different from that in the AB group (*p* = 0.015, *R* = 0.892) and FMT group (*p* = 0.006, *R* = 0.548), while the bacterial population structures in the samples from the AB and FMT groups (*p* = 0.314, *R* = 0.012) were not significantly different. These findings suggest that FMT or antibiotic treatment could change the composition of the microbiota (Figure 7). The UniFrac distance, determined based on the unweighted pair group method with arithmetic mean (UPGMA) further revealed different abundant bacterial types among the groups (Figure 5 and Supplementary 4).

**TABLE 2:** The multi-response permutation procedures difference analysis of the four groups.

Group	Distance	A	Observed_delta	Expect_delta	*P*-value	P_adj_BH
FMT-AB	Bray-Curtis	0.386155973	0.389498482	0.634523535	0.01	0.01
FMT-Con	Bray-Curtis	0.536033148	0.269322769	0.580478470	0.009	0.01
FMT-DC	Bray-Curtis	0.377307031	0.478107294	0.767805833	0.01	0.01
AB-Con	Bray-Curtis	0.416252491	0.358614904	0.614332222	0.008	0.01
AB-DC	Bray-Curtis	0.290814886	0.567399430	0.800072391	0.008	0.01
Con-DC	Bray-Curtis	0.406858798	0.447223716	0.753991992	0.008	0.01

#### Bacterial composition difference analysis

Next, we analyzed the gut microbiota composition of these groups. Significant differences at the phylum level were observed between the groups. The composition at the phylum level is shown in a histogram (Figure 3). Firmicutes and Bacteroidetes were shown to be the most abundant bacteria among these groups. The proportion of Firmicutes in the FMT group was significantly higher than that in the Con group (*p* < 0.01) but was significantly lower than that in the AB group (*p* < 0.005). The proportion of Bacteroidetes in the FMT group was significantly higher than that in the AB group (p < 0.01), but not than that in the Con group (Figure 3). The Firmicutes/Bacteroides (F/B) ratio in the FMT group was not significantly different from that in the Con group (Figure 3). Interestingly, the F/B ratio (0.85) in the FMT group was significantly lower than that in the AB group (F/B = 856.2).

**Figure 8:**
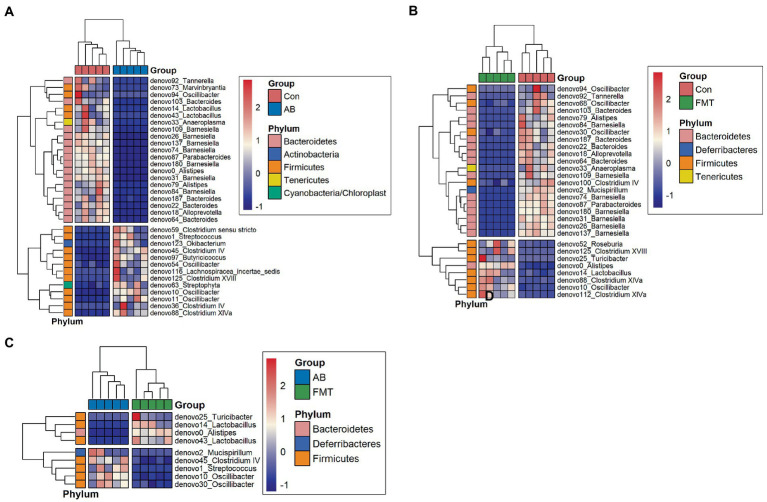
Heatmap of differential species abundance clustering between Con and AB groups **(A)**, Con and FMT groups **(B)**, and AB and FMT groups **(C)**. The heatmap shows the hierarchical clustering of samples based on the relative abundance of fecal microbiota in the FMT, AB and Con groups. The relative values in the heatmap (after normalization), depicted by color, indicate the degree of aggregation or content of bacterial species among samples at the genus level. The color gradient from blue to red indicates low to high relative abundance, respectively. The vertical clustering indicates the similarity in the richness of different species among different samples. The closer the distance between two species, the shorter the branch length, indicating greater similarity in richness between the two species. Horizontal clustering indicates the similarity of species richness in different samples. Similarly, the closer the distance between two samples, the shorter the branch length, indicating greater similarity in richness of species between the two samples. Con, Control group; FMT, fecal microbiota transplantation group; AB, antibiotic group.

At the genus level (Figure 8), there were 29 genera that differed significantly between the Con and FMT groups; among these, the proportions of 21 were significantly higher in the Con group, while the proportions of the others were significantly higher in the FMT group (Figure 8C). In addition, 34 genera showed significant differences between the Con and AB groups; the proportions of 21 of these were significantly higher in the Con group, while the proportions of 13 were significantly higher in the AB group (Figure 8B). Interestingly, significant differences in the proportions of 9 genera were observed between the AB and FMT groups; the proportions of 4 (*denovo25_Turicibacter, denovo14_Lactobacillus*, *denovo0_Alistipes*, and *denovo43_Lactobacillius*) were significantly higher in the FMT group, while the proportions of 5 (*denovo2_Mucispirillum*, *denovo45_Clostridium IV*, *denovo1_Streptocossus*, *denovo10_Oscillibacter*, and *denovo30_Oscillibacter*) were significantly higher in the AB group (Figure 8A). In this panel, we found the abundance of *Mucispirillum* was decreased in FMT group when compared to AB and Con groups (Figures 8B, C), while there was not significantly difference between AB group and Con group (Figure 8A). This was indicated that decreased of the abundance of *Mucispirillum* was not effect by the antibiotics but by the FMT. Based on the result, a possible explanation might be that the decreased of *Mucispirillum* in the FMT group maybe effect by the FMT from *S. murinus* but bot the antibiotics.

**Figure 9:**
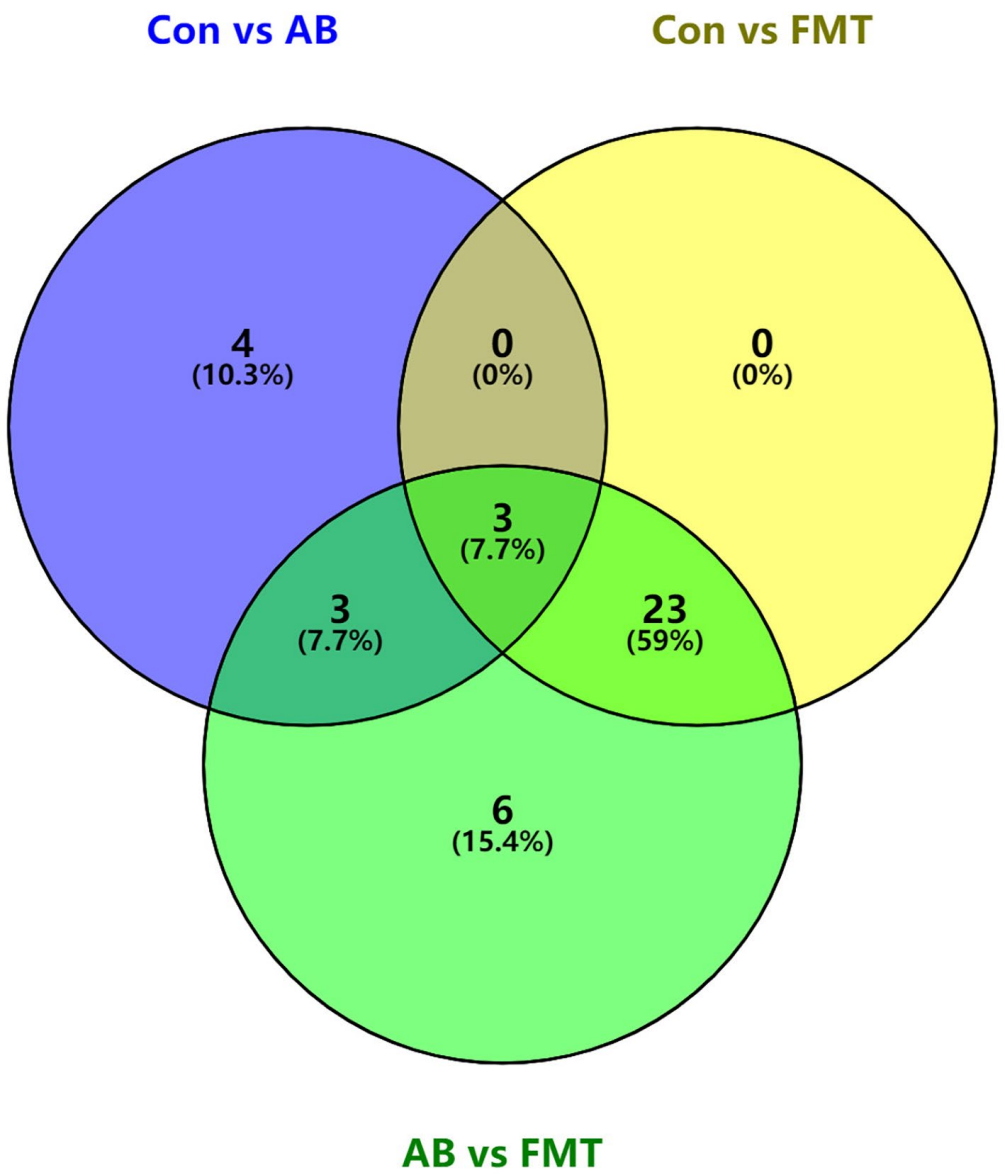
Venn diagram of shared and unique taxa at the genus level among Con, AB and FMT groups. The numbers of shared and unique taxa in the three pairwise comparison groups are shown based on the operational taxonomic units. Con, Control group; FMT, fecal microbiota transplantation group; AB, antibiotic group; DC, donor group.

Then the common bacteria in these groups were also analyzed using a Venn diagram, the Venn diagram (Figure 9) showed three common elements in “Con vs. AB,” “Con vs. FMT” and “AB vs. FMT,” which comprise *denovo0_Alistipes*, *denovo10_Oscillibacter*, *denovo14_Lactobacillus*. Four elements were exclusively included in “Con vs. AB.” Six elements were exclusively included in “AB vs. FMT.” There were 3 common elements in “Con vs. AB” and “AB vs. FMT,” which comprise *denovo1_Streptococcus*, *denovo43_Lactobacillus*, *denovo45_Clostridium IV*. There were 23 common elements in “Con vs. FMT” and “AB vs. FMT.” These analyses indicted that FMT from *S. murinus* or antibiotic treatment has an effect on the bacterial composition of the mouse gut.

### Effect of FMT on gut microbiota

To assess the effect of FMT on the gut microbiota of recipient mice, the differences of gut microbiota were analyzed. The analyses demonstrated that fewer species were observed in the FMT group in comparison to the Con and AB groups (Figure 2) under the same sequence numbers. The Chao1 and Shannon diversity indexes (Figure 6) also showed that the microbiota diversity and richness in the FMT group were lower in comparison to the Con and AB groups. This indicated, that FMT has an effect on the gut microbiota. The PCoA (Figure 7) showed that the structure of the microbiota in the FMT group was significantly changed (Con vs. FMT, *R* = 0.548) in comparison to the Con group. Intriguingly, the structure of the microbiota in the FMT group was similar to that of the DC group (FMT vs. DC, *R* = 0.260). At the genus level (Figure 8C), *denovo0*_*Alistipes*, *denovo14*_*Lachnospiraceae*, *denovo10*_*Oscillibacter* was more abundant in the FMT group than in the Con group, while the abundance of *denovo92*_*tannerella*, *Oscillibacter* (*denovo68*, *denovo30*), *Bacteroides* (*denovo103*, *denovo187*, *denovo64*, *denovo22*), *denovo79*_*Alistipes*, *denovo18*_*Alloprevotella*, *denovo33*_*Anaeroplasma*, *denovo100*_*Clostridium IV*, *denovo2*_*Mucispirillum*, *denovo87*_*Parabacteroides*, *Barnesiella* (*denovo180*, *denovo31*, *denovo26*, *denovo137*, *denovo74*, *denovo109*, *denovo84*) in the FMT group was lower in comparison to the Con group.

Effect of antibiotic administration on the gut microbiota

Following antibiotic treatment alone, a large change in the microbiota was found at the genus level (Figure 4). As it showed, the number of observed species in the AB group was lower in comparison to the Con group but higher in comparison to the FMT group (Figure 2) under the same sequences’ numbers. The Chao 1 and Shannon diversity indexes (Figure 6) also showed that the microbiota diversity and richness in the AB group were lower in comparison to the Con but higher in comparison to the FMT group. This indicated that antibiotics had an effect on the gut microbiota. The principal coordinate analysis (Figure 7) revealed that the structure of the microbiota in AB group (Con vs. AB, *R* = 0.892) was significantly changed in comparison to the Con group and the UPGMA tree (Figure 5 and Supplementary 4) showed consistent results to those described above. At the genus level, 34 genera showed significant differences between the Con and AB groups; 21 of these were significantly higher in the Con group and 13 were significantly higher in the AB group. In comparison to the Con group, the AB group showed a significant increase in 21 genera, and a significant decrease in 13 genera (Figure 8B). All of this indicated that broad-spectrum antibiotics had an effect on the gut microbiota.

## Discussion

The present study was conducted to test whether the gut microbiota can be transferred from *S. murinus* to SPF mice and to explore the potential function of the gastrointestinal microbiota of *S. murinus*. In this study, 16S rRNA-amplicon sequencing was used to perform a comprehensive analysis of the gut microbiota in *S. murinus* and C57BL mice. High-throughput pyrosequencing as well as an operational taxon-based analysis showed significant changes in the gut microbial communities between groups of mice. Statistically significant differences in alpha diversity were observed among the FMT, AB and Con groups. Furthermore, the beta diversity analysis indicated an apparent structural separation among the FMT, AB and Con groups, indicating that FMT or antibiotic treatment may affect the overall composition of intestinal flora.

The changes of the body weight and blood lipid level of mice were measured, no significant changes were observed in the body weight between the AB and FMT groups, in blood lipid level between the FMT group and other groups. However, there was a difference in body weight between the AB group and the FMT group versus the Con group, and it persisted to the end. As shown in Supplementary 2, the body weight of the AB and FMT groups dropped after antibiotics were started, and then began to rise after the antibiotics were stopped, while the body weight of the Con group that did not use antibiotics during this period showed a natural increase without a process of decline. However, although the body weight of the AB and FMT groups began to recover and increase after the withdrawal of the drug, it still did not return to the weight level of the Con group, and until the end of the experiment, the weight difference between the two groups and the Con group did not continue to increase or decrease, but remain unchanged. These indicated that the weight difference between the AB and FMT groups and the Con group was not due to the transplantation itself, but due to the side effects of antibiotics on the gastrointestinal tract, which affected normal eating, resulting in temporary weight loss. Moreover, it also implies that the feeding of the AB group and FMT groups was no longer affected without the influence of antibiotics (after drug withdrawal), and the body weight continued to increase just like the Con group. The transplantation of *S. murinus* intestinal flora with obesity-resistant properties into SPF mice did not lead to changes in the body weight or blood lipid level of the SPF mice.

The structure and composition of *S. murinus* gut microbiota were analyzed in the present study. The microbiota of *S. murinus* was enriched in Firmicutes (75.55%) and Proteobacteria (21.07%), while Bacteroidetes were not detected, which is consistent with a previous study (Shinohara et al., 2019). Proteobacteria phyla was enriched in *S. murinus*, which rarely exists in rodents. Proteobacteria was found to be the most predominant phylum in the giant panda and is related to lignin digestion as well as the catabolism of various components (Fang et al., 2012; Mukhopadhya et al., 2012). We inferred that the higher relative abundance of Firmicutes and Proteobacteria observed in the present study might be related to its obesity-resistant properties.

In this study, fewer OTUs were detected in the intestine gut of *S. murinus* in comparison to the mouse groups. Ley et al. (2008) reported that fecal microbiota diversity in mammals was high in herbivores, low in carnivores, and intermediate in omnivores. Although, we did not obtain definitive evidence, it is speculated that the low diversity of the microbiota might be related to the morphological features of the gastrointestinal tract and *S. murinus* as an insectivore (Shinohara et al., 2019). Our previous studies (Yi et al., 2010, 2011) demonstrated that *S. murinus* lack fermentative chambers, such as a forestomach and cecum. These characteristics may limit the physiological space and physical area where microbes occur. For example, Bacteriodetes, such as *Bacteroidaceae*, *Prevotellaceae* and *Rikenellaceae* are often found in the cecum (Donaldson et al., 2016). As *S. murinus* lack a cecum, these bacteriodetes was not detected in the gut of *S. murinus* in this study.

*Suncus murinus* has been used in various fields of science due to its unique characteristics, which are distinct from laboratory rodents, including as models of emesis (Ebukuro et al., 2000), *in vivo* motilin studies (Tsutsui et al., 2009), and as a natural obesity-resistant animal model in recent studies (Yi et al., 2010; Zhang et al., 2020); however, the mechanism of natural obesity resistance is still not well understood. In this study, lactic acid bacteria (*Enterococcus*, *Lactococcus*, *Streptococcus*, and *Vagococcus*) were abundant in the gut of *S. murinus* at the genus level, implying that lactic acid fermentation might be important in *S. murinus*. As revealed in human studies (Pessione, 2012), lactic acid bacteria contribute to the complexity of the gut microbiota and have many beneficial effects on metabolism and immunomodulation. It should be noted that these lactic acid bacteria and other members of Firmicutes are not the core members of the human or mouse gut microbiota (Nguyen et al., 2015), indicating that the gut microbiota in *S. murinus* is quite different from that in humans and mice.

In the present study, the FMT group was found to have lower gut microbiota diversity and richness in comparison to the Con group, we hypothesize that the lower diversity of the gut microbiota in the FMT group was closely related to the donor *S. murinus*. Interestingly, the alpha diversity analysis showed that the diversity and richness in the FMT group was also significantly lower than that in the AB group. When we compared the alpha diversity between the AB and Con groups, the AB group was found to be significantly less rich in comparison to the Con group, while there was no significant difference in diversity between the Con and AB groups. It should be noted that a past study reported that most microbial communities recovered 4 weeks after antibiotic treatment, but some remained reduced after 6 months (Dethlefsen and Relman, 2011).

From the analysis of phylum, the Firmicutes and Bacteroidetes were the prominent phyla in both the FMT and Con groups. While Bacteroidetes normally existed in the FMT (54.46%) and Con (55.89%) groups, Bacteroidetes only was fewer (0.1%) detected in the AB group. That indicated that the Bacteroidetes could not be kill completely by the antibiotics. We speculate that Bacteroidetes might be very sensitive to antibiotics, and most of them were wiped out by antibiotics. However, after receiving FMT, it was re-established due to the strength of *S. murinus* intestinal bacteria, that is to say, FMT, may provide an environment to help Bacteroidetes re-emerge in recipients. Consistent with a previous study (Zhang et al., 2017), we found that antibiotic treatment resulted in an increased abundance of Firmicutes and a decreased abundance of Bacteroidetes and that FMT treatment significantly reversed the increase in the F/B ratio in mice. These findings suggest that FMT from *S. murinus* can suppress the growth of Firmicutes and may further affect the immune function (Ashraf and Shah, 2014).

To investigate the differences in the flora among each experimental group in detail, the bacteria were analyzed at the genus level. At the genus level, the relative abundance of *Alistipes*, *Lachnospiraceae* was significantly higher in the FMT group, while the abundance of *Tannerella*, *Oscillibacter*, *Bacteroides*, *Alloprevotella*, *Anaeroplasma*, *Clostridium IV*, *Mucispirillum*, *Parabacteroides*, and *Barnesiella* were significantly lower in the FMT group in comparison to the Con group. Among the three groups in the present study, the relative abundance of major pathogen genera was lowest in the FMT group. *Mucispirillum,* which was specific and which rarely exist in humans, was enriched in Con mice (Li et al., 2018). Interestingly, *Mucispirillum* was not observed in the FMT group but was observed in the AB and Con groups. This indicated that the gut of the FMT group was different from that of the Con group or AB group. This may have been caused by the transplantation of the intestinal flora of *S. murinus* in FMT. *Lactobacillus*, a well-known probiotic, was depleted after antibiotic treatment. In this study, FMT significantly increased the abundance of *Lactobacillus*, indicating that FMT is able to maintain homeostasis of the gut microbiota by enriching *Lactobacillus*. Ashraf and Shah (2014) also reported that FMT treatment not only restored the intestinal barrier but also increased the abundance of *Lactobacillus*. Moreover, *Lactobacillus* is associated with restoration of the balance of the intestinal immune system.

From the results, we found that the mice could benefit from receiving FMT from *S. murinus*, while the effects of antibiotics in the study cannot be ignored. When we compared the FMT and AB groups, significant differences in 9 genera were observed between the AB and FMT groups, among which *Turicibacter*, *Lactobacillus*, *Alistipes*, and *Lactobacillius* were significantly more abundant in the FMT group while *Mucispirillum*, *Clostridium IV*, *Streptocossus*, and *Oscillibacter* (*denovo10* and *denovo30*) were significantly higher in the AB group. Twenty-one genera were found to be significantly more abundant in the Con group, while 13 genera were significantly more abundant in the AB group. That is, there were large changes in the composition and structure of the gut flora in the AB group in comparison to Con group, suggesting a special role of antibiotics in the experimental study of intestinal flora transplantation (Ramirez et al., 2020). This was consistent with the results of the alpha and beta diversity analyses. The results demonstrated a high microbiota diversity and richness in the AB group in comparison to the FMT group. We speculate that antibiotics can suppress the bacteria for a short time and that in most cases, the bacteria would recover after the withdrawal of antibiotics. These results are consistent with previous studies (Dethlefsen and Relman, 2011).

Although the use of antibiotics has no obvious side effects on the host, it could be cause significant changes in the gut microbiota, resulting in varying degrees of impact on health (Dethlefsen and Relman, 2011). In line with this, in our study, the Con group showed a high microbiota richness in comparison to the AB and FMT groups, while the Con group showed no difference microbiota diversity in comparison to the AB group. It should be reminded that a reduction in the overall number of bacteria does not imply a reduction in bacterial diversity (Hou et al., 2022). As antibiotic-sensitive bacteria are largely reduced or eliminated, antibiotic-resistant bacteria will multiply and replace the killed bacteria. In fact, even with reduced species diversity, the total microbial load may increase after antibiotic treatment (Panda et al., 2014).

In our study, a cocktail of antibiotics (ampicillin, vancomycin, neomycin, and metronidazole) was used to deplete the gut microbiota before the FMT procedure. Broad-spectrum antibiotic treatment can decrease the bacterial load by multiple orders of magnitude in 2 weeks of treatment or less (Brown et al., 2017). This was consistent with our study. Individual antibiotics can be used to shift the composition of the gut microbiota in order to identify classes of bacteria that are relevant to different phenotypes (Schubert et al., 2015). Due to differences in the mechanism of action, antibiotics can selectively deplete different members of the microbiota. For example, ampicillin, decreases bacterial diversity and increases the prevalence of *Enterobacter* spp., metronidazole and clindamycin both target anaerobes and decrease the overall diversity, vancomycin is only effective against Gram-positive bacteria and decreases bacterial diversity, and polymyxin B specifically targets Gram-negative bacteria (Atarashi et al., 2011; Schubert et al., 2015). In past literature reports, researchers have used a variety of regimens that differ in the combination and doses of antibiotics and duration of treatment (Supplementary 1). All of these combinations broadly target Gram-positive, Gram-negative, and anaerobic bacteria. However, the utilization of antibiotics not been standardized.

The detrimental effects of antibiotics on the microbiota must be further considered when using antibiotics for pre-treatment for fecal microbiota transplantation. Although the use of broad-spectrum antibiotics has been shown to cause changes in the fecal microbiota of healthy individuals, many gastrointestinal studies did not discuss the antibiotic exposure of the host and understanding the effects of antibiotics on the host should be a prerequisite for study design (Wang et al., 2018). With the development of modern sequencing methods, we can gain a more comprehensive understanding of the impact of antibiotics on microbial communities, thereby bringing new vitality to the use of antibiotics (Hou et al., 2022). Therefore, when choosing an antibiotic, we need to conduct further experiments to evaluate its effectiveness.

In conclusion, the innovative features of this experiment was that we used a natural obesity-resistant experimental animal (*S. murinus*) for the transplantation of gut bacteria. C57BL mice that received the gut microbiota of *S. murinus* showed significant changes in gut microbial composition and bacterial diversity. Although FMT from *S. murinus* failed to completely colonize the intestinal tract of the mice, it still had a certain effect on the establishment of the intestinal flora of the mice. However, in comparison to the analysis of the results after FMT, the significant impact of antibiotics on transplantation itself should be prioritized as a research topic. Only when the use of antibiotics before transplantation is standardized will it be possible to objectively analyze the results of transplantation.

## Data availability statement

The datasets presented in this study can be found in online repositories. The names of the repository/repositories and accession number(s) can be found in the article/Supplementary material.

## Ethics statement

The animal study was reviewed and approved by the Animals: house musk shrews (*Suncus murinus*) and C57BL/6NCr mice.

## Author contributions

MZ, SQ-Y, and HS acquired funding and designed and conceived the study. MZ and TY performed the experiments. MZ, SQ-Y, MH, CY, JL, JC, and RL analyzed the data. MZ wrote the manuscript. All authors contributed to the final version of the manuscript, read, and approved the final manuscript.

## Funding

This work was supported by a Grant-in-Aid for Scientific Research from the Ministry of Education, Culture, Sports, Science and Technology of Japan (No. 19K07271).

## Conflict of interest

The authors declare that the research was conducted in the absence of any commercial or financial relationships that could be construed as a potential conflict of interest.

## Publisher’s note

All claims expressed in this article are solely those of the authors and do not necessarily represent those of their affiliated organizations, or those of the publisher, the editors and the reviewers. Any product that may be evaluated in this article, or claim that may be made by its manufacturer, is not guaranteed or endorsed by the publisher.

## Footnotes

^1^http://weizhong-lab.ucsd.edu/cd-hit-otu

^2^https://www.r-project.org/
