# Supplementary material for: Fecal microbiota transplantation from Suncus murinus, an obesity-resistant animal, to C57BL/6NCrSIc mice, and the antibiotic effects in the approach
Source: Front Microbiol. 2023 Apr 6;14:1138983. doi: 10.3389/fmicb.2023.1138983 (PMC10117937; doi:10.3389/fmicb.2023.1138983)
Supplement: Supplementary file 1 [file Data_Sheet_1.docx]

Supplementary Material

**Fecal Microbiota Transplantation from *Suncus murinus,* an Obesity-Resistant Animal, to C57BL/6NCrSIc Mice**

Mingshou Zhang^1^, Hiraku Sasaki^2^, Ting Yang^1^, Juefei Chen^1^, Rujia Li^1^, Cheng Yi^3^, Jun Li^4^, Maozhang He^5^, Shuang-Qin Yi^1^*

**Correspondence to:

Shuang-Qin Yi, M.D., Ph.D., Professor

Department of Frontier Health Sciences, Graduate School of Human Health Sciences, Tokyo Metropolitan University, 7-2-10, Higashiogu, Arakawa-ku, 116-8551 Tokyo, Japan. Email: [yittmniu@tmu.ac.jp](mailto:yittmniu@tmu.ac.jp)

Tel.: +0081-3-3819-1211

# Supplementary Data

**Supplement Legends**

**Supplement 1**

**References of Supplement 1**

Adami, A. J., Bracken, S. J., Guernsey, L. A., Rafti, E., Maas, K. R., Graf, J., et al. (2018). Early-life antibiotics attenuate regulatory T cell generation and increase the severity of murine house dust mite-induced asthma. *Pediatr. Res*. 84, 426–434. doi: 10.1038/s41390-018-0031-y

Amorim, N., McGovern, E., Raposo, A., Khatiwada, S., Shen, S., Koentgen, S., et al. (2022). Refining a Protocol for Faecal Microbiota Engraftment in Animal Models after Successful Antibiotic-Induced Gut Decontamination. *Front. Med.* 9, 770017. doi: 10.3389/fmed.2022.770017

Atarashi, K., Nishimura, J., Shima, T., Umesaki, Y., Yamamoto, M., Onoue, M., et al. (2008). ATP drives lamina propria T(H)17 cell differentiation. *Nature* 455, 808–812. doi: 10.1038/nature07240

Chen, Y., Zhang, S., Zeng, B., Zhao, J., Yang, M., Zhang, M., et al. (2020). Transplant of microbiota from long-living people to mice reduces aging-related indices and transfers beneficial bacteria. *Aging* 12, 4778–4793. doi: 10.18632/aging.102872

Ericsson, A. C., Personett, A. R., Turner, G., Dorfmeyer, R. A., and Franklin, C. L. (2017). Variable Colonization after Reciprocal Fecal Microbiota Transfer between Mice with Low and High Richness Microbiota. *Front. Microbiol*. 8, 196. doi: 10.3389/fmicb.2017.00196

Freitag, T. L., Hartikainen, A., Jouhten, H., Sahl, C., Meri, S., Anttila, V. J., et al. (2019). Minor Effect of Antibiotic Pre-treatment on the Engraftment of Donor Microbiota in Fecal Transplantation in Mice. *Front. Microbiol.* 10, 2685. [doi: 10.3389/fmicb.2019.02685](https://doi.org/10.3389/fmicb.2019.02685)

Gudi, R., Suber, J., Brown, R., Johnson, B. M., and Vasu, C. (2020). Pretreatment with Yeast-Derived Complex Dietary Polysaccharides Suppresses Gut Inflammation, Alters the Microbiota Composition, and Increases Immune Regulatory Short-Chain Fatty Acid Production in C57BL/6 Mice. *J. Nutr.* 150, 1291–1302. doi: 10.1093/jn/nxz328

Josefsdottir, K. S., Baldridge, M. T., Kadmon, C. S., and King, K. Y. (2017). Antibiotics impair murine hematopoiesis by depleting the intestinal microbiota. *Blood* 129, 729–739. doi: 10.1182/blood-2016-03-708594

Khosravi, A., Yáñez, A., Price, J. G., Chow, A., Merad, M., Goodridge, H. S., et al. (2014). Gut microbiota promote hematopoiesis to control bacterial infection. *Cell Host Microbe* 15, 374–381. doi: 10.1016/j.chom.2014.02.006

Kinnebrew, M. A., Ubeda, C., Zenewicz, L. A., Smith, N., Flavell, R. A., and Pamer, E. G. (2010). Bacterial flagellin stimulates toll-like receptor 5–dependent defense against vancomycin-resistant enterococcus infection. *J. Infect. Dis*. 201, 534–543. doi: 10.1086/650203

Luo, S., Zhang, H., Jiang, X., Xia, Y., Tang, S., Duan, X., et al. (2023). Antibiotics administration alleviates the high fat diet-induced obesity through altering the lipid metabolism in young mice. *Lipids* 58, 19–32. doi: 10.1002/lipd.12361

Saxena, A., Moran, R. R. M., Bullard, M. R., Bondy, E. O., Smith, M. F., Morris, L., et al. (2022). Sex differences in the fecal microbiome and hippocampal glial morphology following diet and antibiotic treatment. *PLoS One*. 17, e0265850. doi: 10.1371/journal.pone.0265850

Shinohara, R., Nakashima, H., Emoto, T., Yamashita, T., Saito, Y., Yoshida, N., et al. (2022). Gut Microbiota Influence the Development of Abdominal Aortic Aneurysm by Suppressing Macrophage Accumulation in Mice. *Hypertension* 79, 2821–2829. doi: 10.1161/HYPERTENSIONAHA.122.19422

Sun, J., Xu, J., Ling, Y., Wang, F., Gong, T., Yang, C., et al. (2019). Fecal microbiota transplantation alleviated Alzheimer's disease-like pathogenesis in APP/PS1 transgenic mice. *Transl. Psychiatry* 9, 189. doi: 10.1038/s41398-019-0525-3

Su, H., Liu, J., Wu, G., Long, Z., Fan, J., Xu, Z., Liu, J., Yu, Z., Cao, M., Liao, N., Peng, J., Yu, W., Li, W., Wu, H., & Wang, X. (2020). Homeostasis of gut microbiota protects against polychlorinated biphenyl 126-induced metabolic dysfunction in liver of mice. *Sci. Total Environ.* 720, 137597. doi: 10.1016/j.scitotenv.2020.137597

Wang, S., Huang, M., You, X., Zhao, J., Chen, L., Wang, L., et al. (2018). Gut microbiota mediates the anti-obesity effect of calorie restriction in mice. *Sci. Rep*. *8*, 13037. doi: 10.1038/s41598-018-31353-1

Yan, J., Herzog, J. W., Tsang, K., Brennan, C. A., Bower, M. A., Garrett, W. S., et al. (2016). Gut microbiota induce IGF-1 and promote bone formation and growth. Proc. *Natl. Acad. Sci. U.S.A*. 113, E7554–E7563. doi: 10.1073/pnas.1607235113

**Supplement 2.** The body weight curves of FMT group (FMT), AB group (AB) and Con group (Con). Abx, the days (from day 8 to day 17) of antibiotic treatment; FT, the days (from day 20 to day 22) of FMT. “✢” means *P* < 0.01

**Supplement 3.** Comparisons of the phospholipids (PL), total cholesterol (T-Cho) and triglycerides (TG) in blood serum lipids level among groups before FMT (A, B, C) and after FMT (D, E, F). The same letter indicates that there was no significant difference between the groups; a different letter indicates a statistically significant difference between the groups. Con, Control group; FMT, fecal microbiota transplantation group; AB, antibiotic group.

**Supplement 4.** Cluster analysis with the Unifracdistance matrix (unweighted Unifrac distance). A column of numbers on the right side is the code of the subjects in each experimental group. Con group: 115, 116, 118, 119 and 121; AB group: 108, 109, 110, 113 and 114; FMT group: 101, 104, 106, 107 and 111; DC group: N.DC1~N.DC5.

# Supplementary Figures and Tables

For more information on Supplementary Material and for details on the different file types accepted, please see [here](https://www.frontiersin.org/guidelines/author-guidelines#supplementary-material).

## Supplementary Figures

**
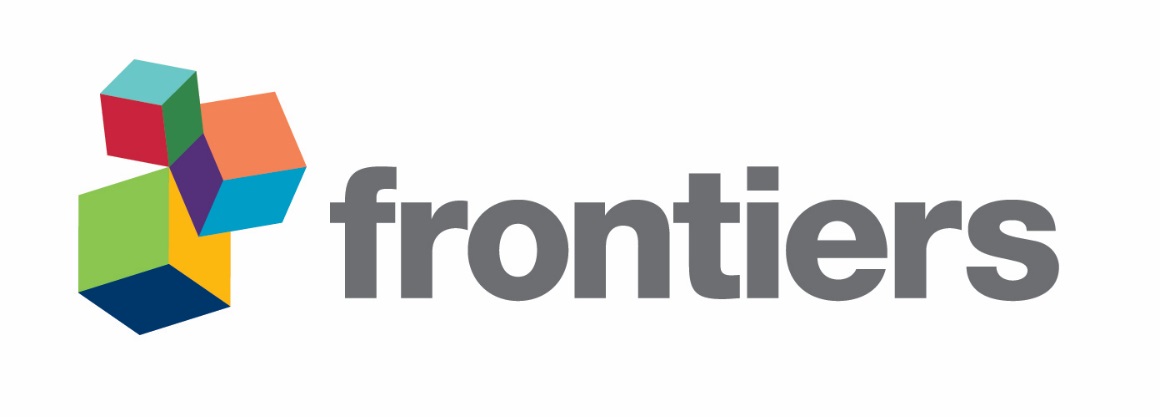
**
