# Supplementary figures and images for: Fecal microbiota transplantation from Suncus murinus, an obesity-resistant animal, to C57BL/6NCrSIc mice, and the antibiotic effects in the approach
Source: Front Microbiol. 2023 Apr 6;14:1138983. doi: 10.3389/fmicb.2023.1138983 (PMC10117937; doi:10.3389/fmicb.2023.1138983)

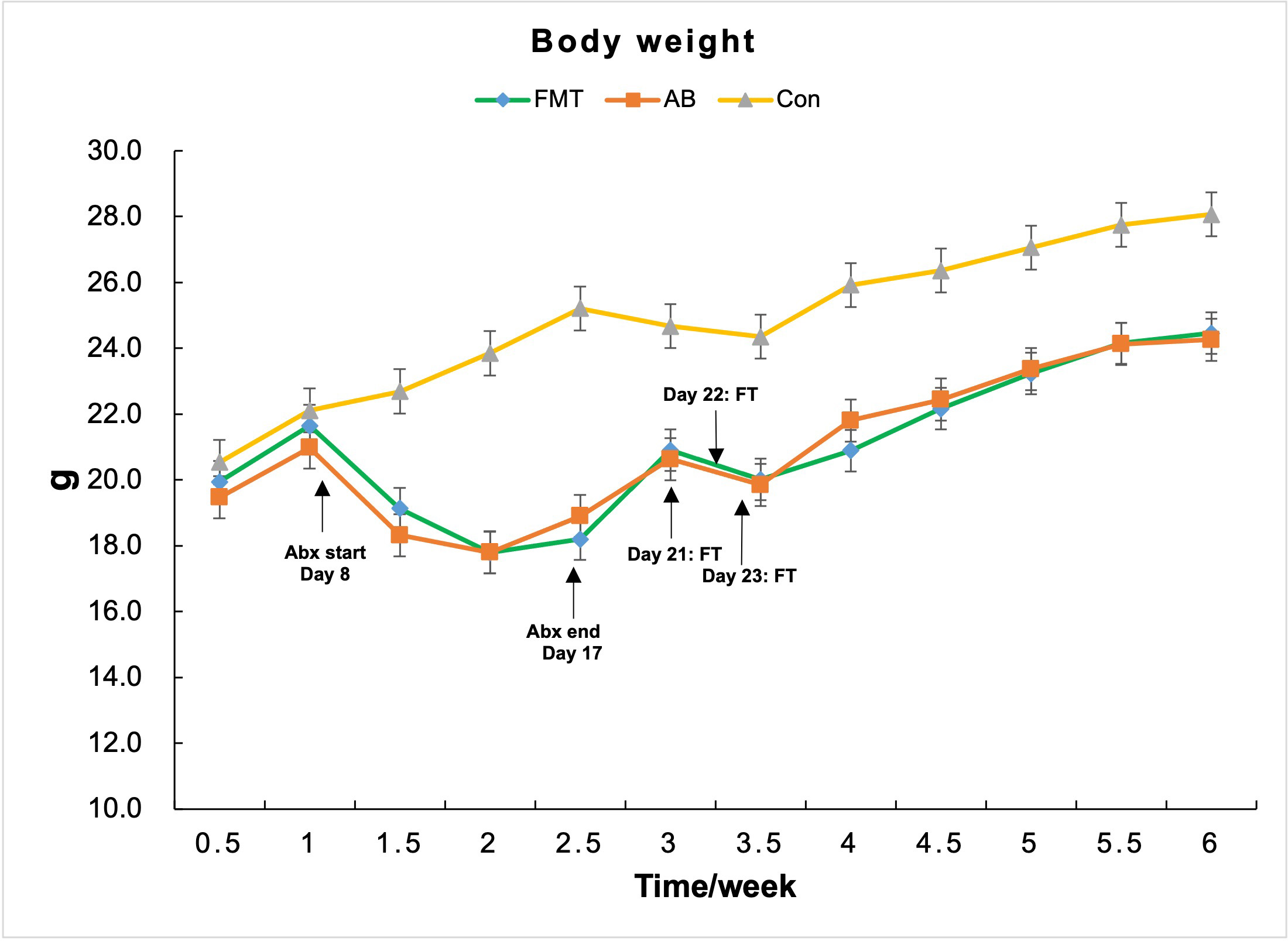

Supplement: Supplementary file 2 [file Image_1.JPEG]

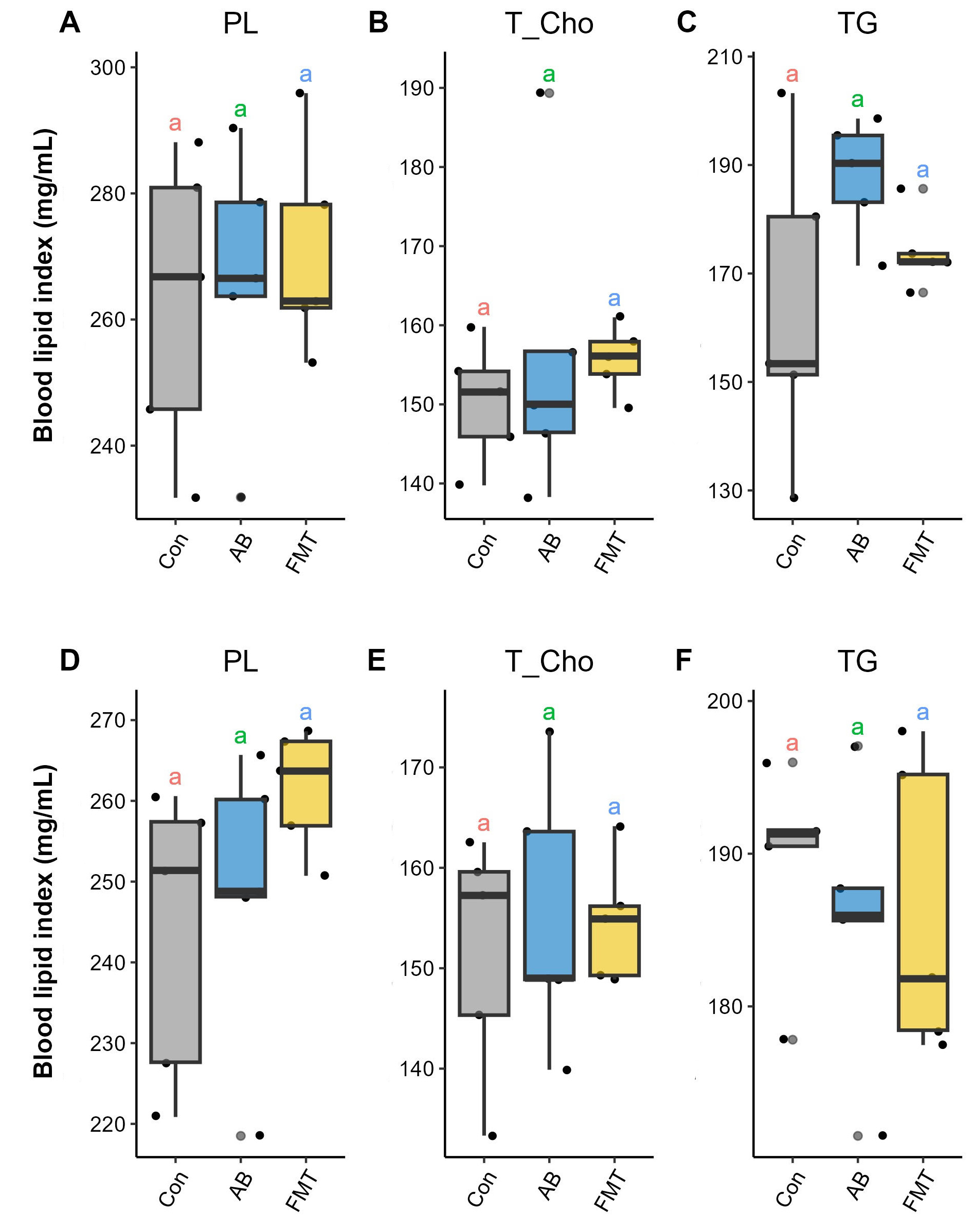

Supplement: Supplementary file 3 [file Image_2.JPEG]

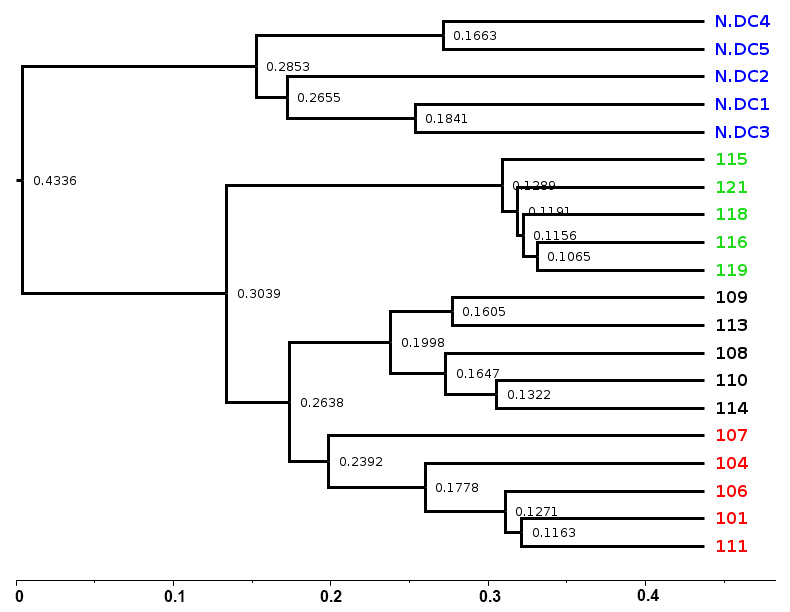

Supplement: Supplementary file 4 [file Image_3.JPEG]
